# Reply to D. O'Reilly et al

**DOI:** 10.1200/GO.21.00077

**Published:** 2021-05-06

**Authors:** Amol Patel, Vineet Govinda Gupta, Bivas Biswas, Sandip Ganguly, Chandan K. Das, Atul Batra, Sainath Bhethanabhotla

**Affiliations:** Amol Patel, MD, DM, Army Hospital Research & Referral, New Delhi, India; Vineet Govinda Gupta, MD, DM, Artemis Hospitals, Gurugram, India; Bivas Biswas, MD, DM; and Sandip Ganguly, MD, DM, Tata Medical Center, Kolkata, India; Chandan K. Das, MD, DM, Post Graduate Institute of Medical Education and Research, Chandigarh, India; Atul Batra, MD, DM, All India Institute of Medical Sciences, New Delhi, India; and Sainath Bhethanabhotla, MD, DM, Care Cancer Institute, Hyderabad, India

We thank O'Reilly et al^1^ for their letter responding to our article. We feel that a Fulvestrant-Big Mac Index is an interesting way to compare cancer drug affordability between nations, since the price of a Big Mac offers a rough idea of purchasing power parity, with some limitations.^2^ We would like to suggest some ways to improve the index, if possible.

First, although fulvestrant is a good comparator, being a drug that is off-patent and commonly used around the world, its absence from the WHO Model List of Essential Medicines^3^ may represent a limitation for wider comparison between developing and developed countries. It may help the applicability of the index to use an off-patent drug from the WHO list. Second, we suggest that monthly salary (a subjective measurement that may be unreliable) may be replaced with per capita income or gross domestic product, an objective measurement that is tracked by economic agencies (eg, World Bank). For example, Figure 1 indicates that the monthly salary in Pakistan is $5,000 in US dollars, equal to the United States, which is far from the truth—the per capita gross domestic product of Pakistan is $1,284 in US dollars and that of United States is more than 50 times that of Pakistan.

We agree whole-heartedly that the high cost of cancer treatment is not just a third-world problem. Irrespective of the wealth of a nation, money spent on cancer drugs is money not spent on other health or development needs. In the United Kingdom's National Health Service, the National Institute for Health and Care Excellence evaluates the cost-effectiveness of new drugs. This is one way of checking on high price of cancer drugs in publicly funded health systems. The same may not be applicable in a country like India, where the majority of treatment is offered through out-of-pocket spending. A few recent publications have indicated that even commonly used drugs like trastuzumab and temozolomide are not cost-effective in India at currently used protocols, despite the lower cost.^4,5^ An index like the Fulvestrant-Big Mac may allow a comparison of cost of drugs between various countries and help identify areas for policy intervention.
